# Determinants of cervical cancer screening utilization among women in Southern Ethiopia

**DOI:** 10.1038/s41598-022-18978-z

**Published:** 2022-09-01

**Authors:** Samuel Yohannes Ayanto, Tefera Belachew, Muluemebet Abera Wordofa

**Affiliations:** 1 Department of Population and Family Health, Jimma University, Jimma, Ethiopia; 2 Department of Nutrition and Dietetics, Jimma University, Jimma, Ethiopia

**Keywords:** Cancer, Health care, Oncology

## Abstract

Cervical cancer has been an important public health problem. Despite the availability of screening services, its utilization in Ethiopia is low. This study therefore, aimed to identify contextual predictors of cervical cancer screening utilization among eligible women. This study employed facility-based unmatched case–control study design. Data were collected from 410 participants using interviewer-administered techniques. The collected data were entered using EpiInfo version 7 and transported to SPSS version 20 for statistical analysis. We performed descriptive analysis and logistic regression to identify predictors of screening utilization. This study demonstrated that urban residence, being in marital union, membership in women development army, knowledge of cervical cancer screening location, use of maternal health care in the previous year and knowledge on cervical cancer and its screening were predictors of screening utilization. Therefore, it is important to improve women’s knowledge on cervical cancer, promote maternal health care use, disseminate health information through women’s groups and consider all positive effects of urban residence among rural women to improve screening utilization.

## Introduction

Precancerous cervical lesions develop whenever there is persistent infection with Human Papilloma Virus (HPV). When treatment is delayed, the lesions progress to advanced cancerous stage^1^. Global Cancer Observatory (GLOBOCAN) estimated nearly 530,000 cases and 275,000 deaths caused by cervical cancer worldwide in 2008 of which less developed world accounted for 85% of the disease burden^2^. In 2012, there were an estimated 527,600 new cases and 265,700 deaths due to cervical cancer worldwide^3^. Of this, 85% of new cases and 87% of deaths took place in low- and middle-income countries (LMICs)^4^. In 2018, nearly 570 000 women were affected and 311 000 died from the disease worldwide^5^.

In Sub-Saharan Africa (SSA), cervical cancer had the highest incidence^3^ and prevalence rates^6^ and appeared to be the second most common cancer of reproductive women in the region in 2012^7^. Southern and Eastern Africa shared the highest burden of the disease^5,8^. Moreover, Eastern Africa shared the highest mortality rate due to cervical cancer in 2018^9^. World Health Organization (WHO) estimated that cervical cancer will cause 443,000 deaths in 2030 globally^10^. Of these, 98% will occur in low income countries, with SSA sharing the highest burden^4^.

Despite this statistics, its incidence and mortality have been substantially lowered in high resource countries during the last few decades primarily due to the implementation of screening packages and the availability of improved treatment options^11^. However, in low and middle income countries cervical cancer remains a significant public health problem^9^.

In Ethiopia, a trend analysis of cancer registry data from Tikur Anbesa Specialized Hospital (TASH), showed that 5293 cervical cancer cases were diagnosed between 1997 and 2012 which accounted for 31.8% of all new cases of cancer^12^. In 2015 it also accounted for about 20% of all identified female cancer cases in the country^13^. Nearly 6,300 new cases and 4,884 deaths due to cervical cancer occurred in Ethiopia each year^8^.

The Ethiopia Federal Ministry of Health (FMOH) developed national comprehensive cervical cancer prevention and control guideline to reduce the disease burden and defined eligible women (30–49 years) for cervical screening across the country. The ministry has been committed to avail effective screening methods in health care facilities for women in the target group^14^. Even though more recent studies in the country reported screening uptake of 9.9–15.5%^15–17^ still it is lower than 80% screening target set nationally for women in the 30–49 years by 2020^14,18^.

Studies have shown that availability of effective screening programs and its utilization can lead to a significant reduction in the morbidity and mortality associated with advanced cervical cancer disease^11^. Despite the importance and its availability, only few of eligible women underwent screening for cervical cancer in Ethiopia. This implies that different contextual factors might have contributed to this low level of cervical cancer screening utilization among women in the target group.

In this regard, different studies have been conducted in Ethiopia to identify factors that could have affected cervical cancer screening utilization in different settings. Accordingly, different contextual factors that could have determined screening utilization have been identified. These included occupation^19–21^ having multiple sexual partnership^20,22–24^, information or knowledge about cervical cancer^19–24^ knowing someone who have ever screened^21^, health service providers’ advice and counseling^19,21,25^, perceived disease susceptibility^21,23^, history of STI^22,23,25^, perceived barriers^23^, education^22^, visits to gyneacological unit and family planning use^19^, age at first sexual experience^24^, participant’s age, positive attitude towards screening, history of visit to health institution, and family history of cervical cancer^25^.

But most of these studies employed crosectional study design which is inherently weak to reliably identify predictors of screening utilization that can be regarded for implementation purposes. This calls for further research works to identify predictors using strong study designs to provide planners with reliable evidence. Our study therefore, aimed to identify predictors of cervical cancer screening utilization using case–control study design among eligible women in Southern Ethiopia.

## Methods

### Study setting

We conducted this study in Kembata Tembaro and Hadiya zones which are located in the Southern Nations Nationalities and Peoples' Regional State, Ethiopia. The earlier zone had a total population of 941,311 of which females constituted 464,146 in 2020 and 41,158 women were eligible for cervical cancer screening. Whereas the later zone had a total population of 1,815,110 of which females represented 917,175 and 81,200 women were eligible for cervical cancer screening in 2020. The study was conducted in health facilities which had been identified to have provided cervical cancer screening servives in the zones. Accordingly, Nigist Eleni Mohammed Memorial Comprehensive Specialized Hospital, Hosanna health center, Shurmo health center, Dr. Bogalech General hospital, Mudula Primary hospital and Doyogena Primary hospital were included in our study. The study was conducted from October to November 2021 using facility based unmatched case–control study design.

### Participants’ selection

The study included women in the study area who are eligible for cervical cancer screening according to the recommendations contained in the national cervical cancer prevention and control guideline. Generally, the eligible women’s age ranges from 30 to 49 years. Accordingly, cases and controls were selected in similar time frames from those women who visited cervical cancer screening and family planning service units respectively in the selected health facilities. Those women who were eligible for cervical cancer screening and received the screening service were recruited for the case category and those eligible women who have not received the screening service within the five years period were selected for the control category.

Cases were women who received cervical cancer screening service during the study period and within the five years period whereas controls were eligible women who visited health facility for other health care services but have not received cervical cancer screening within the last five years and during the study period. Sample was allocated to each health facility based on the existing caseload in the cervical screening units of the respective health facilities. Cases were successively recruited and included in the study until the required sample size has been saturated whereas controls were selected parallel to the case recruitment process.

### Sample size determination

The sample size for the study was determined using the following double population proportion formula^26^,

$$n = \left( {\frac{r + 1}{r}} \right)\frac{{\left( {\overline{p}} \right)\left( {1 - \overline{p}} \right)\left( {Z_{\beta } + Z_{\alpha /2} } \right)^{2} }}{{\left( {{\text{p}}_{1} - p_{2} } \right)^{2} }}.$$

Accordingly, the proportion of exposed among case group (p1) 52.9%, the proportion of exposed among control group (p2) 38.8%^27^, the ratio of sample size in case group to sample size in control group (r) 1:1, the desired level of statistical significance (α) 5% and the value of the normal distribution at 5% significance level (Zα/2) 1.96 and the desired power (80%) of the study 0.84 were used. Based on these assumptions, our sample size per study group was 196 women i.e. 196 women in the case group and 196 women in the control group. Therefore, n = 392 women. To adjust for incomplete responses, 5% has been added to the sample size which yielded 412 study participants.

### Data collection and instrument

Data were collected using structured questionnaire designed from relevant literature sources for the purpose of meeting the research objectives. The data collection instrument was pretested to check for its clarity, logical sequence, and language appropriateness etc. and subsequent modification was made accordingly. Also, data were collected by interviewing the selected women in the private counseling rooms in their respective health facilities. Data collectors and supervisors were trained about the data collection process. The completed questionnaire was checked for its consistency and completeness by the supervisors during the entire data collection period. All methods were performed in accordance with the relevant guidelines and regulations.

### Operational definition and measurement

#### Knowledge

Twenty items on cervical cancer and its screening were delivered to study participants with ‘yes’ or ‘no’ options. Each correct response was given a score of 1 and a wrong answer given a score of 0. We checked the twenty knowledge assessment items for their internal consistency using reliability test. Consequently, once we confirmed that the reliability is within the acceptable range, we computed the mean score to determine the overall knowledge of the study participants on cervical cancer and its screening and classified as having poor and good knowledge based on the mean score value.

#### Attitude

We assessed the attitude using a Likert’s scale using three options i.e. disagree = 1, neutral = 2 and agree = 3. The responses were summed up and a total score was obtained for each participant. Then, we calculated the mean score for each participant. We also checked the ten attitude assessment items for their internal consistency using reliability test and we excluded two poorly correlated items from the analysis. Finally, we calculated the mean attitude score and considered those who scored the mean and above value as having favorable attitude, whereas those women who scored below the mean value were categorized as having unfavorable attitudes towards cervical cancer & its screening.

### Data management and analysis

The data were entered in to Epi info version 7 and transported to SPSS version 20 for statistical analysis. Frequencies and percentages of different variables were computed for description of the data as appropriate. Bivariable logistic regression analysis was done to identify potentially candidate predictors. Those predictor variables which were significantly associated at p value less than 25% in bivariable analysis were taken in to multivariable logistic regression. Then multivariable logistic regression with 95% confidence interval was carried out to assess the association between cervical screening utilization and the candidate predictor variables. Odds ratio with confidence interval was determined to see the strength of the association and the significance level was declared at p value less than 5%.

### Ethics approval and consent to participate

This study has been approved by the Institutional Review Board of Jimma University institute of health with approval letter number IHRPGn/355 on 16/7/2021. Written permission was also obtained from the respective administrative hierarchies of the health sector including the health facilities. Each participant was assured of the confidentiality of the information they provided and consented immediately before data collection to ensure voluntary participation in the study using the consent format. Informed consent was obtained from all the study subjects.

## Results

### Sociodemographic characteristics

A total of 410 women participated in our study which represents 99.5% response rate of which 50.2% were cases and 49.8% were controls. The mean age of the study participants in the case group and control group was 35 ± 4 and 36 ± 4 years respectively. Most of the study participants, 205 (99.5%) of the cases and 178 (87.3%) of the controls, were in a marital union. Protestant Christianity is the major religion among the study participants where 142 (68.9%) of the cases and 127 (62.3%) of the controls belong to. The two major ethnic groups among the study participants were Hadiya and Kembata. Thus, 102 (49.5%) of the study participants in the case group and 93 (45.6%) of the study participants in the control group belong to Hadiya Ethnicity whereas 64 (31.1%) of the study participants in the case group and 60 (29.4%) of the study participants in the control group belong to Kembata Ethnicity. The major occupation of the study participants was household roles which accounted 133 (64.6%) in the case group and 129 (63.2%) in the control group (Table 1).

**Table 1:** Socio-demographic characteristics of study participants in Hadiya and Kembata Tembaro zones, SNNPRG, Ethiopia, October to November 2021.

Back ground variable	Variable categories	Case n (%)	Control n (%)
Residence (n = 410)	Urban	129 (62.6)	94 (46.1)
	Rural	77 (37.4)	110 (53.9)
Current marital status (n = 410)	Currently in union	205 (99.5)	178 (87.3)
	Not in union	1 (0.5)	26 (12.7)
Religion (n = 410)	Protestant	142 (68.9)	127 (62.3)
	Islam	10 (4.9)	16 (7.8)
	Orthodox	38 (18.4)	43 (21.1)
	Catholic	15 (7.3)	13 (6.4)
	Others*	1 (0.5)	5 (2.5)
Ethnicity (n = 410)	Hadiya	102 (49.5)	93 (45.6)
	Kembata	64 (31.1)	60 (29.4)
	Tembaro	23 (11.2)	34 (16.7)
	Others**	17 (8.3)	17 (8.3)
Participant’s age (n = 410)	30–39	179 (86.9)	162 (79.4)
	40–49	27 (13.1)	42 (20.6)
Participant’s age at marriage (n = 410)	< 18 years	32 (15.5)	26 (12.7)
	± 18 years	174 (84.5)	178 (87.3)
Participants’ education (n = 410)	No formal education	23 (11.2)	27 (13.2)
	Primary education (1–8)	97 (47.1)	112 (54.9)
	Secondary education (9–12)	47 (22.8)	44 (21.6)
	Tertiary education (12 +)	39 (18.9)	21 (10.3)
Participant’s occupation (n = 410)	Household roles	133 (64.6)	129 (63.2)
	Employee	37 (18)	26 (12.7)
	Merchant	28 (13.6)	36 (17.6)
	Others***	8 (3.9)	13 (6.4)
Occupation of the husband (n = 383)	Employee	59 (28.8)	44 (24.7)
	Merchant	68 (33.2)	65 (36.5)
	Farmer	64 (31.2)	58 (32.6)
	Others****	14 (6.8)	11 (6.2)
Education of the husband (n = 383)	No formal education	5 (2.4)	12 (6.7)
	Primary education (1–8)	87 (42.4)	80 (44.9)
	Secondary education (9–12)	60 (29.3)	48 (27)
	Tertiary education (12 plus)	53 (25.9)	38 (21.3)
Monthly income (n = 410)	First quartile	43 (20.9)	78 (38.2)
	Second quartile	49 (23.8)	35 (17.2)
	Third quartile	55 (26.7)	47 (23)
	Fourth quartile	59 (28.6)	44 (21.6)
Family size (410)	≤ 4.6	110 (53.4)	99 (48.5)
	> 4.6	96 (46.6)	105 (51.5)
Parity (410)	≤ 4.15	167 (81.1)	162 (79.4)
	> 4.15	39 (18.9)	42 (20.6)

Others* Adventist, Apostolic; Others** Amhara, Wolayita; Others *** Student, Daily laborer, Farmer; Others **** Student, Daily laborer; Not in union refers to single, widowed, divorced and separated.

The study participants were majorly engaged in to marital relations at the age of 18 years and above among which 174 (84.5%) were cases and 178 (87.3%) were controls. Cases and controls constitute 43 (20.9%) and 78 (38.2%) in the first quartile whereas 59 (28.6%) and 44 (21.6%) in the fourth quartile of monthly family income respectively. The family size of 110 (53.4%) of the study participants among the cases and 99 (48.5%) of the study participants among the controls have less than or equal to 4.6 persons per household. One hundred sixty seven (81.1%) of tha cases and 162 (79.4%) of the controls have achieved parity level which is less than or equal to 4.15 deliveries (Table 1).

### Participant’s awareness and information sources

Among the study participants, 203 (99.5%) of the cases and 75 (36.4%) of the controls had been informed about cervical cancer and screening services before the study period. Health workers were the main channels or sources of information regarding cervical cancer and its screening which constituted 149 (73%) for the case group and 28 (13.6%) for the control group. Among the study participants, 63 (30.6%) of the cases and 61 (29.9%) of the controls knew other women in their social network who underwent cervical cancer screening before. Also 20 (9.7%) of the participants in the case group and 19 (9.3%) of the participants in the control group knew other women who had been diagnosed with cervical cancer.

One hundred fifty nine (77.2%) and 106 (52%) of the study participants in the case and control groups respectively had been aware of the service locations where cervical cancer screening services were provided. Only 55 (26.8%) of the cases and 50 (28.1%) of the controls had had discussion with their husbands regarding cervical cancer and its screening (Table 2).

**Table 2:** Awareness level and information sources among study participants in Hadiya and Kembata Tembaro zones, SNNPRG, Ethiopia, October to November 2021.

Variable name	Variable categories	Case n (%)	Control n (%)
Informed of cervical cancer and its screening (n = 410)	Yes	203 (99.5)	75 (36.4)
	No	1 (0.5)	131 (63.6)
Source of information (n = 278)	Health workers	149 (73%)	28 (13.6)
	Mass media	43 (21.1)	39 (18.9)
	Social network	8 (3.9)	5 (2.4)
	Women development army	3 (1.5)	3 (1.5)
Knew anyone who underwent screening (n = 410)	Yes	63 (30.6)	61 (29.9)
	No	143 (69.4)	143 (70.1)
Knew anyone who had cervical cancer (n = 410)	Yes	20 (9.7)	19 (9.3)
	No	186 (90.3)	185 (90.7)
Knowledge of screening location (n = 410)	Yes	159 (77.2)	106 (52)
	No	47 (22.8)	98 (48)
Discussion with their husbands (n = 383)	Yes	55 (26.8)	50 (28.1)
	No	150 (73.2)	128 (71.9)

### Health service related factors

Our study demonstrated that, 135 (65.5%), of the study participants in the case group and 90 (44.1%) in the control group had been members of women development army (WDA). Also, 99 (48.1%) of cases and 117 (57.4%) of the controls were beneficiaries of health insurance. In majority of the cases, 186 (90.3%), and controls, 163 (79.9%), there existed a family member who visited a health facility in the previous year for health care services. Moreover, majority of the study participants in the case group, 192 (93.2%) and control group, 144 (70.6%) visited health facilities to use maternal health care services in the previous year before the study. Majority of the study participants in both the case group, 146 (70.9%) and the control group, 131 (64.2%) reported that they have lived at 30 min or more walking distance from the nearest health center. Similarly, 149 (72.3%) of the cases and 151 (74%) of the controls resided at 30 min or more walking distance from the nearest hospital. Health extension workers made at least one home visit in majority of the cases, 170 (82.5%) and controls, 155 (76%) in the previous year before the study (Table 3).

**Table 3:** Health service related characteristics among study participants in Hadiya and Kembata Tembaro zones, SNNPRG, Ethiopia, October to November 2021.

Service related characteristics	Variable Categories	Case n (%)	Control n (%)
WDA membership (n = 410)	Yes	135 (65.5)	90 (44.1)
	No	71 (34.5)	114 (55.9)
Health insurance status of the family (n = 410)	Yes	99 (48.1)	117 (57.4)
	No	107 (51.9)	87 (42.6)
Family members’ visit to health facility last year (n = 410)	Yes	186 (90.3)	163 (79.9)
	No	20 (9.7)	41 (20.1)
Use of any maternal health services last year (n = 410)	Yes	192 (93.2)	144 (70.6)
	No	14 (6.8)	60 (29.4)
Walking distance from the nearest health center (n = 410)	< 30 min	60 (29.1)	73 (35.8)
	≥ 30 min	146 (70.9)	131 (64.2)
Walking distance from the nearest hospital (n = 410)	< 30 min	57 (27.7)	53 (26)
	≥ 30 min	149 (72.3)	151 (74)
Number of home visits made by HEWs last year (n = 410)	No visits	36 (17.5)	49 (24)
	At least 1 visits	170 (82.5)	155 (76)

### Participants’ knowledge

Two hundred seventy six (67.3%) of the study participants have had correct responses about multiple sexual partnership being a risk factor for the development of cervical cancer. The proportion of the study participants who scored correct answer regarding the cause of cervical cancer being human papilloma virus was 227 (55.4%). Items concerning signs and symptoms of cervical cancer were generally the least correctly answered items of all knowledge assessment items. Accordingly, spotting between periods and foul smelling discharge were symptoms correctly identified by 191 (46.6%) and 227 (55.4%) participants respectively. Also, pain during sexual intercourse and heavy menses were accounted for by 187 (45.6%) and 91(22.2%) participants respectively as symptoms of cervical cancer.

Two hundred seventy one (66.1%) of the study participants recognized that cervical cancer is preventable disease. Regarding the methods of prevention, 258 (62.9%) of them identified early screening and treatment as a mechanism for cervical cancer prevention. Also, 217 (52.9%) and 145 (35.4%) of the study participants correctly identified risk reduction and vaccination as methods for cervical cancer prevention respectively. Only 186 (45.4%) of the study participants correctly responded to the item stating about the age eligibility of women for cervical cancer screening in the Ethiopian context.

Majority of the study participants correctly identified the advantages of early screening for cervical cancer which included early detection of the disease, 212 (51.7%), early treatment options, 230 (56.1%), and prevention of advanced disease conditions, 212 (51.7%). Only 155 (37.8%) of the study participants correctly answered the item regarding the five years’ regular screening interval for cervical cancer. In summary, 128 (62.1%) of the cases and 97 (47.5%) of the controls scored the mean (10.56) value and above and had been considered as having good knowledge regarding cervical cancer and its screening.

### Participants’ attitude

The major proportion of study participants, 244 (59.5%), showed agreement with an item specifying that every woman is at risk of developing cervical cancer in her life time. One hundred ninety two (46.8%) of the study participants disagreed with the statement demonstrated that women do not need to be screened for cervical cancer if they feel healthy. One hundred eighty eight (45.9%) of the study participants did not agree with the incurability of cervical cancer. Major percentage of the study participants, 319 (77.8%), demonstrated an agreement with the item suggesting that cervical cancer is preventable.

Two hundred eighty eight (70.2%) participants agreed that getting screened for cervical cancer benefits women even when they feel healthy. Two hundred thirty four (57.1%) of the study participants showed agreement regarding attitude item specifying that women’s busyness with household roles should not hinder them get screened for cervical cancer. Generally the participants’ mean attitude score was 2.34 ± 0.48 on three Likert’s scale ranging from 1 to 3. Accordingly, 100 (48.5%) of the participants in the case group and 96 (47.1%) of the participants in the control group have been regarded as having favorable attitude towards cervical cancer and its screening.

### Determinants of cervical cancer screening utilization

Binary logistic regression was performed to ascertain the effect of independent variables on the likelihood that participants underwent screening for cervical cancer. For this purpose, variables which showed association with cervical cancer screening utilization at p-value 0.25 were transported to multivariable logistic regression. The logistic regression model was statistically significant at, x^2^ (11) = 144, *p* < 0.001 based on Omnibus test of model coefficients and fits the data well, x^2^ (8) = 12, *p* = 0.15 in Hosmer and Lemshow goodness of fit test. The model explained 39% (Nagelkerke R^2^) of the variance in cervical cancer screening utilization and correctly classified 74% of the cases.

A multivariable logistic regression showed that, after adjusting for other confounders, urban residence, being in marital union, membership in women development army (WDA), knowledge of cervical cancer screening service location, use of maternal health care services in the previous year and participants’ knowledge on cervical cancer and its screening were statistically significantly associated with cervical cancer screening service utilization. Accordingly, study participants with urban residence were 2.7 times more likely to use cervical cancer screening when compared with their rural counterparts [AOR: 2.7, 95% CI: (1.56, 4.56)]. Similarly, study participants who were in marital union were 26.41 times more likely to use cervical cancer screening as compared to study participants in other marital categories [AOR: 26.41, 95% CI: (3.18, 219.44)].

Study participants who had been members of women development army were 2.65 times more likely to use screening services for cervical cancer when compared to their non-member counterparts [AOR: 2.65, 95% CI: (1.60, 4.39)]. Likewise, participants who knew the location for cervical cancer screening service were 7.49 times more likely to use cervical cancer screening services when compared to those who did not have knowledge on cervical cancer screening locations [AOR: 7.49, 95% CI: (4.10, 13.68)]. Moreover, study participants who utilized maternal health care services in the previous year before the study were 4.88 times more likely to use cervical cancer screening when compared with those participants who did not use maternal health care services in the year [AOR: 4.88, 95% CI: (2.35, 10.17)]. Also participants who scored the mean and above mean in knowledge assessment items were 3.14 times more likely to utilize cervical cancer screening services when compared with those who scored below the mean value.

On the other hand participants’ age, age at marriage, participants’ education and occupation, monthly family income, family member’s visit to health facility in the previous year, attitude, husbands’ education and occupation, distance from health facility, family size, number of live births and other variables were not significantly associated with screening service utilization (Table 4).

**Table 4:** Multivariable binary logistic regression predicting cervical cancer screening utilization among participants in Hadiya and Kembata Tembaro zones, SNNPRG, Ethiopia, October to November 2021.

Variable	Case n (%)	Control n (%)	Crude OR 95%CI	Adjusted OR 95% CI	*P* value
**Participant’s age**					
30–39	179 (86.9)	162 (79.4)	1	1	0.00
40–49	27 (13.1)	42 (20.6)	0.58 (0.34, 0.99)	0.98 (0.50, 1.91)	0.94
**Residence**					
Urban	129 (62.6)	94 (46.1)	1.96 (1.32, 2.91)	2.67 (1.56, 4.56)	0.00
Rural	77 (37.4)	110 (53.9)	1	1	0.00
**Marital status**					
Married	205 (99.5)	178 (87.3)	29.94 (4.02, 222.89)	26.41 (3.18, 219.44)	0.00
Others	1 (0.5)	26 (12.7)	1	1	0.00
**Monthly income**					
1st quartile	43 (20.9)	78 (38.2)	1	1	0.00
2nd quartile	49 (23.8)	35 (17.2)	2.54 (1.43, 4.50)	0.98 (0.48, 1.98)	0.95
3rd quartile	55 (26.7)	47 (23)	2.12 (1.24, 3.64)	1.19 (0.61, 2.31)	0.61
4th quartile	59 (28.6)	44 (21.6)	2.43 (1.42, 4.17)	0.76 (0.37, 1.59)	0.47
**Membership WDA**					
Yes	135 (65.5)	90 (44.1)	2.41 (1.62, 3.59)	2.65 (1.60, 4.39)	0.00
No	71 (34.5)	114 (55.9)	1	1	0.00
**Know screening location**					
Yes	159 (77.2)	106 (52)	3.13 (2.04, 4.79)	7.49 (4.10, 3.68)	0.00
No	47 (22.8)	98 (48)	1	1	0.00
**Families visited HF last year**					
Yes	186 (90.3)	163 (79.9)	2.34 (1.32, 4.16)	1.15 (0.52, 2.56)	0.73
No	20 (9.7)	41 (20.1)	1	1	0.00
**Use of maternal services**					
Yes	192 (93.2)	144 (70.6)	5.71 (3.07, 10.63)	4.88 (2.35, 10.17)	0.00
No	14 (6.8)	60 (29.4)	1	1	0.00
**Participants’ knowledge**					
Poor knowledge	78 (37.9)	107 (52.5)	1	1	0.00
Good knowledge	128 (62.1)	97 (47.5)	1.81(1.22, 2.68)	3.14 (1.79, 5.50)	0.00

## Discussion

The Federal Ministry of Health Ethiopia, in its strategic documents, recommended regular cervical cancer screening every five years and at least once for every woman in her life time. In resource-poor settings, including Ethiopia, 30–49 years old women are the target beneficiaries for the current recommendations and screening services. Cervical cancer has been commonly identified within this age range in the country and rare event in women under the age of 30 years except for high-risk groups^14,18^. This age group has been considered appropriate for the identification of the predictors of screening uptake, implementation of effective screening and treatment services. To benefit women from the importance of early screening and treatment, identification of its predictors paves fertile ground for further implementation of effective interventions. In this study, different predictors of utilization of cervical screening have been identified.

Our study showed that, the utilization of cervical cancer screening services was significantly higher among study participants having good knowledge in knowledge assessment items regarding cervical cancer when compared with those participants who have had poor knowledge. Similarly, studies conducted in Ethiopia, Arbaminch^28^, Mekele^23^, Shebedino^22^, Diredawa^19^ and Tanzania^29^ demonstrated that participants’ good knowledge on cervical cancer is significantly associated with screening services utilization as compared to participants’ with poor knowledge. Generally, it has been established that knowledge on particular issues reinforces desired attitude and promotes positive health behavior among target individuals^30^. Therefore, the observed participants’ good knowledge in our study could have positively contributed to the desired participants’ behavior to utilize the screening services more.

In this study it was found that knowledge on the location where the cervical cancer screening has been provided was one of the significant predictors of cervical cancer screening utilization. This finding is consistent with the finding reported by the study conducted in rural Uganda^31^. Cues to action are important factors which may play positive role in the development of desired health behavior. Being aware of the service location as cue to action in addition to knowledge on cervical cancer and screening could have helped study participants to get screened for cervical cancer more in this group.

The proportion of participants screened in the age group 30–39 years was higher when compared to the proportion of participants screened in the age group 40–49 years. This shows that among the study participants who were screened, majority of them received the services in the age group 30–39 years as compared to age group 40–49 years. However, unlike other studies, this is not significantly associated with cervical cancer screening utilization. Different studies demonstrated that cervical screening service utilization was significantly higher in the age group from 30–39 to 40–49 years when compared with those below 30 years^20,23,29^. Our study has included only participants who are eligible for screening with in the national context, age 30–49 years, and did not include participants in the younger age group who are below 30 years. This limitation restricted us from making comparison between our study participants and younger age groups.

Though, it was not statistically significant, screening service utilization in our study vary across participants’ occupational statuses. That is, majority of the study participants who had an employment opportunity utilized the screening services more when compared to participants with other occupational categories. Other studies conducted in Ethiopia Tigray region and Jimma town demonstrated that screening utilization was significantly higher among employed participants as compared to other occupations^17,20^. This could be due to the fact that employed participants could have gained better opportunities to information exposure and awareness outside the home environment on different health matters. Moreover, in majority of the cases employed participants have higher educational status than unemployed participants which might have contributed to the observed differences in screening practices.

The place of residence was significantly associated with cervical cancer screening utilization as study participants who lived in urban areas were more likely to have been screened than rural participants in our study. Study conducted in Uganda revealed similar findings^31^. Access to cervical screening services may be more difficult in rural settings than urban due to screening centers not being within walking distances, lack of transport and cost of transportation^29,32^. Moreover, urban settings could have provided better health information exposure opportunities to residents due to accessibility of information sources relative to rural settings which could have brought differences in screening utilization between urban and rural residents.

In our study, participants who had been members of women development army (WDA) utilized cervical cancer screening significantly more than those who were not members of women development army. This formally organized women’s social development army is believed to expose group members to different health related information and creates conducive learning social environment. Additionally, the existing functional link between health extension workers and these teams severs as a channel to circulate and disseminate different information relevant to social welfare including health matters. This could have influenced members in women development army significantly to utilize cervical screening services more.

Our study showed that the use of maternal health care services in the previous year among the study participants significantly predicted the use of cervical cancer screening service. Studies conducted in Debre Markos Ethiopia^25^, Jamaica^33^ and Kenya^34^ reported similar findings in that participants who visited health facilities in previous year for any reason were more likely to utilize cervical screening services than those who did not utilize health care services. Any visit to health care facility provides a good opportunity to receive health education and increases the chances of exposure of service users to health information which would in turn increases health awareness. This could have positively influenced participants’ health behavior to utilize screening services for cervical cancer more.

### Limitations

Our study did not consider participants’ HIV and STIs status for the inclusion or exclusion of participants from the study. Cognizant of the fact that those women who are at risk of HIV and STIs visit health facilities more as compared to the general women population, they could have utilized cervical screening more during the study period. As a result, there could have been high possibility of participants with HIV and STIs to be recruited in the case category with subsequent reduction of the chance of recruitment of women from the general population as cases in to our study. Therefore, this can be considered as the limitation when interpreting the findings of our study.

## Conclusions

This study revealed important predictors for cervical cancer screening utilization among the study participants. These include urban residence, being in marital union, membership in women development army (WDA), knowledge of cervical cancer screening location, use of maternal health care services in the previous year and knowledge regarding cervical cancer and its screening. Based on the findings of our study we recommend concerned parties in the health sector to exercise strategic efforts aimed at improving women’s knowledge on cervical cancer and promoting activities that target the utilization of maternal health care services among the target groups. It is also highly valuable to consider social groupings like women development army and marital union as a channel for health information dissemination activities to realize improved screening service uptake among the groups. Moreover, it is important to consider all positive effects of urban residence in local developmental efforts among rural women in a way that promotes cervical screening utilization.

## Data Availability

Data that will be generated in the research process will be made available after the completion of the research reports through the corresponding author upon requests and appropriate public repositories.

## Abbreviations

Dr.: Doctor
FMOH: Federal Ministry of Health
GLOBOCAN: Global Cancer Observatory
HEWs: Health extension workers
HPV: Human papilloma virus
LMICs: Low- and middle-income countries
SNNPRG: South Nations Nationalities and People Regional Government
SSA: Sub Saharan Africa
TASH: Tikur Anbesa Specialized Hospital
WCBA: Women of child bearing age
WHO: World Health Organization
